# Differential characteristics of young and midlife adult users of psychotherapy, psychotropic medications, or both: information from a population representative sample in São Paulo, Brazil

**DOI:** 10.1186/s12888-015-0651-2

**Published:** 2015-10-29

**Authors:** Sergio L. Blay, Gerda G. Fillenbaum, Erica T. Peluso

**Affiliations:** Department of Psychiatry, Federal University of São Paulo, (Escola Paulista de Medicina - UNIFESP), R. Borges Lagoa, 570, CEP 04038-020 São Paulo, SP BRAZIL; Center for the Study of Aging and Human Development, Duke University Medical Center, Durham, NC USA; Universidade Anhanguera, Rua Maria Cândida, 1.813, CEP: 02071-013 São Paulo, SP Brazil

**Keywords:** Adults, Epidemiology, Common mental disorders, Psychotherapy, Psychotropic medication

## Abstract

**Objective:**

While the personal characteristics of users of psychotherapy and/or psychotropic medications have been examined, direct user comparison of these treatment approaches appears to be rare. Our aim is to ascertain extent of receipt of these services, and identify basic distinguishing characteristics of users.

**Methods:**

Information on demographics, lifetime and past 12 month use of mental health services, and presence of common mental disorders (CMD), was gathered in 2002 using a multi-stage sampling procedure that yielded a population-representative, community-resident sample (*N* = 2000, age 18–65) for São Paulo, Brazil. Analysis used descriptive statistics and logistic regression.

**Results:**

Overall, 9.3 % reported receiving psychotherapy and/or psychotropic medication, 54.3 % of whom did not meet CMD criteria. Of those meeting criteria for CMD (*n* = 455, 22.8 %), 2.9 % reported only psychotherapy, 10.1 % reported only psychotropic medication, and 5.7 % reported both. CMD was associated with use of psychotropic medication (psychotropic medication alone, Odds Ratio (OR) 3.58, 95 % CI 2.33–5.52; together with psychotherapy, OR 4.17, 95 % CI 2.34–7.44). CMD was not associated with use of psychotherapy. Users’ distinguishing characteristics were: psychotherapy only—not married; psychotropics only—increasing age, female, not married; using both—only CMD status. Neither education nor income was associated with use.

**Conclusions:**

Nearly 10 % of all community residents age 18–65, but less than a fifth of the 23 % with CMD, received psychotherapy and/or psychotropic medication. Non-married status increased odds of all treatment types, but CMD presence increased only odds of psychotropic and combined psychotherapy/psychotropic use, with odds of psychotropic only use increasing with age, and for women. Use was equitable with respect to education and income.

## Background

Findings from multiple epidemiological surveys on the presence and treatment of common mental disorders (CMD) in adults, carried out in both developed and developing countries, typically report a notable prevalence of these conditions, but a serious level of under-treatment [1–8]. Various types of treatment for CMD have been developed, but those most commonly reviewed tend to be psychotherapy (of which there is an extensive variety), psychotropic medication, or combined use.

Multiple studies in developed countries have examined and compared the efficacies of these alternative but overlapping types of treatment [8–13]. There has been interest in identifying where care was received (e.g., in primary care or in other settings) [5, 8] and the demographic characteristics of mental health service recipients often, but not always, persons with higher levels of education or income, women, those divorced or separated [14–20]. With the advent of national health systems, there is also considerable interest in determining equitable use regardless of education or income [3–7, 14–16]. Few studies, however, have tried to determine the readily observable ways in which users of psychotherapy, and psychotropic medication, differ from each other. That is the focus of the present study. We examine this issue within the context of identified need for service, as determined by the presence of CMD, using data from a community-representative sample of adults age 18–65 living in São Paulo, Brazil, a major city in a middle income country.

Since previous studies have shown underuse of psychiatric services by persons with CMD and other psychiatric disorders [5, 6, 20], we anticipated that a larger proportion of this sample would meet criteria for CMD than would report use of psychotherapy or psychotropic medication. Further, given an increasing emphasis on treatment by psychotropic medication, we expected greater use of medication than of psychotherapy, together with a greater likelihood that medication users would meet criteria for CMD, since psychotropic medications are designed to alleviate mental health conditions.

## Method

### Sample

Information comes from a May-June 2002 survey of a representative sample of 18–65 year old community residents of the city of São Paulo, Brazil. Details of the multi-stage sampling procedure have been reported previously [21]. São Paulo is divided into districts. Within each district, census tracts were randomly selected. Then, within each census tract, two blocks were selected at random. Within each block the first household was determined by randomly selected cross streets, after which every fourth household was selected. In each household, one resident aged 18 through 65 whose birthday was closest to the date of interview was chosen. Five interviews (with replacement of a person of same sex and similar age in case of refusal) were conducted within each block, yielding groups of 10 subjects. The resulting distribution was proportional to the populations of the districts, and comparable to the gender and age distribution determined by the year 2000 census for the city of São Paulo. The Ethics Committee of the Federal University of São Paulo (UNIFESP) approved the study. All participants signed informed consent forms.

### Data gathering

Trained interviewers using structured questionnaires gathered information in the home on demographic, mental health, and mental health treatment.

#### Identification of Common Mental Disorders (CMD)

To identify potential cases of CMD, in particular persons with symptoms of depression and anxiety, we administered an extensively used screen, the 12-item General Health Questionnaire (GHQ-12) [22, 23]. GHQ-12 is a screening assessment designed to detect probable psychiatric cases. We did not seek a more nuanced classification since, this being part of an epidemiological survey, brevity was essential. GHQ-12 is a subset of the original 60-item GHQ, prepared by removing items endorsed by the physically ill [23]. Previous investigation of the Brazilian Portuguese translation indicated that performance was not affected by demographic characteristics (sex, age, marital status, income, education, minority status), and that it could identify CMD in primary care and community populations [24]. Each of the 12 items addresses personal status in the last 30 days, and is answered on a 4-point scale. For summary purposes, scores of 0 and 1 were recoded to 0, scores of 2 and 3 were recoded to 1, resulting in a potential scoring range of 0–12. For Brazil, a score of 4 or more indicated the presence of CMD with a sensitivity of 82 % and specificity of 77 % [24], which agrees well with the originally reported findings of 88 and 80 % respectively [23]. Internal reliability (Cronbach’s alpha) of 0.76 or better has been reported [25].

#### Sociodemographic characteristics

The sociodemographic characteristics selected for examination in the present study were those found previously to be associated with use of psychotherapy or psychotropic medications [5, 14–16, 26]. They were age, gender, education (0–7 years/ ≥8 years), income ((high: >U.S.$200/month vs. low: ≤U.S.$200/month) based on Brazilian Association of Market Research Institutes (ABEP) guidelines), whether or not married, employed, or born in São Paulo.

#### Assessment of psychotherapy use

To identify use of psychotherapy, participants were asked “Did you ever participate in treatment or in psychotherapy, such as a professional consultation in which the patient talks with a psychologist or therapist about problems and concerns?” Response categories were: Yes/No/Do not know/No response. Those responding “yes”, indicating any lifetime use, were then asked whether treatment had occurred within the previous 12 months, for how long (months) they had received treatment, whether they were currently undergoing treatment, and if so, for how many months they expected to be in treatment. Unlike the situation for psychotropic medication use, no information was obtained on the provider.

#### Assessment of psychotropic medication use

To ascertain psychotropic medication use, each participant was asked “Have you ever used a medication for anxiety, tension, problems sleeping, depression, mental problem or nervousness?” If “yes”, they were asked whether there had been such use in the last 12 months, and asked to name the medications (free recall). Psychotropic medications were grouped by therapeutic class as: anxiolytics, antidepressants, hypnotics, antipsychotics, mood stabilizers, and phytotherapy. Anticonvulsants (carbamazepine, oxcarbazepine, valproate/divalproex, gabapentin, topiramate, lamotrigine) were considered mood stabilizers when participants indicated use in relation to a psychiatric condition.

#### Provider of psychotropic medications

Participants reporting use of psychotropic medications in the last 12 months were asked to identify the medical specialty of the prescriber, or whether the medication had been recommended and provided by a family member or friend.

#### Participant classification by treatment type

Participants were sorted into four treatment groups: received neither psychotherapy nor psychotropic medication, received psychotherapy only, received psychotropic medication only, received both psychotherapy and psychotropic medication.

#### Statistical analyses

Descriptive statistics (*N*, %, p-values based on chi-square or analysis of variance) were used to characterize the sample. For each treatment group separately, all variables statistically significant in the bivariate analysis for that particular treatment were entered into a logistic regression to determine which characteristics were associated with use of a specific treatment. Any variable significant in any of the three logistic regressions (which predicted psychotherapy only use, psychotropic medication only use, use of both treatments) was then included in a multivariable, polytomous logistic regression, permitting direct comparison of all treatment groups. Descriptive analyses using the dichotomized GHQ-12 were repeated using a trichotomized GHQ-12 score (0/ 1–3/ 4+), a 4-level score (0/ 1/ 2–3/ 4+), and the continuous measure. Because of small sample sizes, logistic regression analyses were repeated using only the trichotomized and continuous measures. While the continuous score indicated greater odds of reporting treatment with increase in score, the categorized analyses suggested that report of treatment was associated with a score of ≥4. Association with demographic characteristics remained essentially the same, regardless of GHQ-12 measure. We report here only findings based on the validated dichotomous measure ascertaining presence/absence of CMD, since from a practical point, they may be the most useful. All analyses were conducted using SPSS-20 software.

## Results

The characteristics of the total sample, and of those meeting criteria for CMD, are given in Table 1. The sample includes a slight preponderance of women (52 %), average age is in the late 30s, 61 % have completed 8 or more years of education, and 29 % are in the higher income category. The majority (59 %) is married and unemployed (68 %), approximately half were born in São Paulo, and 23 % met GHQ-12 criteria for CMD. Those with CMD were more likely to be women, and to have less education.

**Table 1:** Common mental disorders by sociodemographic characteristics (*N* = 2000)

	Total sample	Common mental disorders (CMD)		
	*N* (%)	CMD absent (*N* =1545) *N* (%)	CMD present (*N* =455) *N* (%)	Chi-square (except anova for age) *p* value
Gender				
Female	1035 (51.7)	769 (49.8)	266 (58.5)	.001
Male	965 (48.3)	776 (50.2)	189 (41.5)	
Age				
Mean (sd) (years)	38.52 (13.8)	38.65 (14.0)	38.10 (13.2)	.452
Education				
< 7 years	781 (39.1)	580 (37.5)	201 (44.2)	.011
≥ 8 years	1219 (60.9)	965 (62.5)	254 (55.8)	
Income				
Low income	1413 (70.7)	1086 (70.3)	327 (71.9)	.516
Higher income	587 (29.3)	459 (29.7)	128 (28.1)	
Marital status				
Married	1169 (58.5)	917 (59.4)	252 (55.4)	.131
Not married	831 (41.5)	628 (40.6)	203 (44.6)	
Employed				
No	1365 (68.3)	483 (31.3)	152 (33.4)	.388
Yes	635 (31.7)	1062 (68.7)	303 (66.6)	
Born in São Paulo City				
Yes	991 (49.6)	754 (48.8)	237 (52.1)	.218
No	1009 (50.4)	791 (51.2)	218 (47.9)	

Table 2 provides information on the demographic characteristics and CMD status of all who received any treatment (psychotherapy and/or psychotropic medications, 9.3 %), as well as the type (only psychotherapy, 2.15 %; only psychotropic medications, 4.75 %; both, 2.40 %). Of the 23 % (455/2000) who met GHQ-12 criteria for CMD, 18.7 % (85/455) reported receiving any of these treatments. Of those not meeting GHQ-12 criteria, a smaller percentage (6.5 %) but a larger number (*n* = 101), reported treatment. Of those receiving treatment, 54.3 % did not meet criteria for CMD.

**Table 2:** Sociodemographic characteristics of 12-month users of psychotherapy only, psychotropic medication only, and both^a^

	Treatment by psychotherapy or psychotropic medication *N* = 186 (9.3 %)			Psychotherapy only *N* = 43 (2.15 %)			Psychotropic medication only *N* = 95 (4.75 %)			Both psychotherapy and psychotropic medication *N* = 48 (2.40 %)		
	No (*N* = 1814) *N* (%)	Yes (*N* = 186) *N* (%)	*p*	No (*N* = 1957) *N* (%)	Yes (*N* = 43) *N* (%)	*p*	No (*N* = 1905) *N* (%)	Yes (*N* = 95) *N* (%)	*p*	No (*N* = 1952) *N* (%)	Yes (*N* = 48) *N* (%)	*p*
Gender												
Female	1814 (49.7)	133 (71.5)	.001	1008 (51.5)	16 (37.2)	.143	960 (50.4)	75 (78.9)	.001	1004 (51.4)	31 (64.6)	.072
Male	912 (50.3)	53 (28.5)		949 (48.5)	27 (62.8)		945 (49.6)	20 (21.1)		948 (48.6)	17 (35.4)	
Age (y) Mean (sd)	38.3 (13.9)	41.2 (12.7)	.006	38.6 (13.8)	34.2(11.1)	.036	38.2 (13.8)	45.4 (12.2)	.001	38.5 (13.8)	39.0(12.0)	.809
Education												
0–7 y	715 (39.4)	66 (35.5)	.295	770 (39.3)	11(25.6)	.067	740 (38.8)	41 (43.2)	.400	767 (39.3)	14 (29.2)	.155
≥ 8 y	1099 (60.6)	120 (64.5)		1187 (60.7)	32 (74.4)		1165 (61.2)	54 (56.8)		1185 (60.7)	34 (70.8)	
Income												
High	514 (28.3)	73 (39.2)	.002	569 (29.1)	18 (41.9)	.069	554 (29.1)	33 (34.7)	.237	565 (28.9)	22 (45.8)	.011
Low	1300 (71.7)	113 (60.8)		1388 (70.9)	25 (58.1)		1351 (70.9)	62 (65.3)		1387 (71.1)	26 (54.2)	
Married												
Yes	1089 (60.0)	80 (43.0)	.001	1156 (59.1)	13 (30.2)	.001	1123 (59.0)	46 (48.4)	.042	1148 (58.8)	21 (43.8)	.036
No	725 (40.0)	106 (57.0)		801 (40.9)	30 (69.8)		782 (41.0)	49 (51.6)		804 (41.2)	27 (56.2)	
Employed												
Yes	1253 (69.1)	112 (60.2)	.013	1344 (68.7)	21 (48.8)	.006	1307 (68.6)	58 (61.1)	.123	1332 (68.2)	33 (68.8)	.940
No	561 (30.9)	74 (39.8)		616 (31.3)	22 (51.2)		598 (31.4)	37 (38.9)		620 (31.8)	15 (31.2)	
São Paulo City born												
Yes	891 (49.1)	100 (53.8)	.228	962 (49.2)	29 (67.4)	.018	944 (49.6)	47 (49.5)	.988	967 (49.5)	24 (50.0)	.950
No	923 (50.9)	86 (46.2)		995 (50.8)	14 (32.6)		961 (50.4)	48 (50.5)		985 (50.5)	24 (50.0)	
CMD^b^												
Yes	370 (20.4)	85 (45.7)	.001	442 (22.6)	13 (30.2)	.237	409 (21.5)	46 (48.4)	.001	429 (22.0)	26 (54.2)	.001
No	1444 (79.6)	101 (54.3)		1515 (77.4)	30 (69.8)		1496 (78.5)	49 (51.6)		1523 (78.0)	22 (45.8)	

^a^ *P* value based on chi square test, except for age which is based on analysis of variance

^b^CMD = common mental disorders

Bivariate analyses indicated that, compared to those who received none of these treatments, those who reported receiving any treatment were more likely to be female, older, have higher income, not married, unemployed, and have CMD. Considering specific type of treatment, those reporting psychotherapy alone were younger, not married, unemployed, and more likely to have been born in São Paulo. Those reporting psychotropic medication alone were more likely to be female, older, not married, and have CMD. Finally, those reporting both types of treatment were more likely to have higher income, were not married, and have CMD. Those reporting any prescription medication use were more likely to meet criteria for CMD. Those reporting only psychotherapy did not.

Thirty percent (13/43) of users of psychotherapy only, 52 % (49/95) of psychotropic medication only users, and 54.2 % (26/48) of users of both met criteria for CMD. Neither income nor education distinguished users from nonusers of any of these types of treatment.

Multivariable logistic regressions of the treatment categories individually, using treatment-specific variables (Table 3), indicated that only not being married remained significantly associated with psychotherapy alone; all entered variables (higher age, female, not married, meeting criteria for CMD) were associated with psychotropic medication only use; but only meeting criteria for CMD was associated with use of both treatments.

**Table 3:** Separate multivariable logistic regressions for psychotherapy only users, psychotropic medication only users, and users of both^a^

	Psychotherapy only (*N* = 43)		Psychotropic medication only (*N* = 95)		Both psychotherapy and psychotropic medication (*N* = 48)	
	Odds ratio (95 % CI)	*P* value	Odds ratio (95 % CI)	*P* value	Odds ratio (95 % CI)	*P* value
Age	0.99 (.97–1.02)	.568	**1.05 (1.03–1.06)**	.001	---------------	----------
Female	--------------	--------	**3.35 (2.01–5.58)**	.001	---------------	----------
Not married	**2.53 (1.24–5.13)**	.010	**1.80 (1.17–2.76)**	.008	1.75 (0.98–3.32)	.059
Higher income	--------------	--------	---------------	--------	1.83 (0.87–3.81)	.109
Unemployed	0.56 (0.30–1.04)	.064	---------------	--------	---------------	----------
Born in São Paulo	1.50 (0.74–3.05)	.261	---------------	--------	---------------	----------
CMD^b^	--------------	--------	**3.58 (2.33–5.52)**	.001	**4.17 (2.34–7.44)**	.001

^a^The variables included in analysis are those for which a significant difference was found on bivariate analysis. A variable that was not significantly associated with a particular type of treatment is indicated by a dashed line (---------)

^b^CMD indicates common mental disorders as determined by GHQ-12 score

Bolded values are statistically significant

A final polytomous logistic regression (Table 4), in which the referent indicated receipt of none of the services considered, and the explanatory variables were those found to be significant in any one of the analyses reported in Table 3, confirmed previous findings, but additionally indicated the importance of unmarried status for those reporting both types of treatment.

**Table 4:** Polytomous logistic regression with demographic characteristics and CMD as predictors. Referent is no treatment (*N* = 2000)

	Psychotherapy only (*N* = 43)		Psychotropic medication only (*N* = 95)		Both psychotherapy and psychotropic medication (*N* = 48)	
	Odds ratio (95 % CI)	*P* value	Odds ratio (95 % CI)	*P* value	Odds ratio (95 % CI)	*P* value
Age	0.99 (0.97–1.01)	.305	**1.05 (1.03–1.06)**	.001	1.01 (0.99–1.03)	.269
Female	1.84 (0.97–3.48)	.061	**3.47 (2.08–5.78)**	.001	1.72 (0.94–3.15)	.079
Not married	**3.25 (1.65–6.41)**	.001	**1.91 (1.24–2.94)**	.003	**2.06 (1.13–3.74)**	.018
CMD^a^	1.55 (0.80–3.03)	.196	**3.87 (2.51–5.97)**	.001	**4.47 (2.49–8.00)**	.001

^a^CMD = Common mental disorders

Bolded values are statistically significant

### Prescribers of psychotropic medications

For those providing classifiable responses (92.3 %), the main sources of psychotropic medications were psychiatrists (42.4 %), followed by general practitioners (16.7 %), and neurologists (12.9 %), other specialties played a lesser role. Non-medical providers, prescribing phytotherapy, were used by 16.7 %. Two participants received medications from family or friends. The source of payment for these medications is not known since this information was not obtained. Half the participants receiving psychotherapy used privately paid sources.

## Discussion

Of this community-resident, non-elderly, adult population, 23 % met criteria for CMD. This is within the range of 17.6–29.6 % reported by other studies [1–3, 6, 7, 16]. The wide range probably reflects differences in how CMD was determined, and the age ranges examined. As in other studies, CMD was found to be more common among women and persons with less education [7, 16, 19].

Overall, use in the previous 12 months of any of the three types of treatment was limited. Of the total sample, 9.3 % reported use of psychotherapy and/or psychotropic medications, less than half of whom met criteria for CMD. Of those who met criteria for CMD, only 18.7 % reported receiving any of these treatments. These findings are in agreement with major US and WHO Mental Health Surveys, indicating that in countries at all development levels, a substantial number of users of mental health services do not meet criteria for psychiatric impairment [5, 7, 20]. Reasons for use when psychiatric criteria are not met at time of evaluation vary, and may include prevention, maintenance, successful treatment (CMD no longer present), and test misclassification as unimpaired. While not meeting criteria for a psychiatric diagnosis, many may nevertheless have some indicator of need [27]. It should also be noted that psychotherapy can address concerns that are not necessarily associated with a psychiatric condition or a psychiatric diagnosis (e.g., family, social issues, low level of quality of life, problems with functional ability, history of mental disorder and personality disorder).

People with more serious problems are more likely to get treatment [7, 21], others may report low perceived need, prefer self-management, use non-practitioners, or use approaches available on the internet [26, 28–30]. Some may feel that a stigma is attached, have a negative attitude to mental health services [31–33], or have difficulty accessing services. Additionally, problems may remit (remission rates of 30–54 % have been reported) [8, 29, 34], making a decision to forgo treatment not unreasonable. Alone, neither psychotherapy nor psychotropic medication is effective for all cases, although combined use may be more effective, and important alternative treatments exist [9, 11, 12]. Our treatment rate of 18.7 % may reflect under-treatment, but is not extraordinary, and may not be as disturbing as the number suggests.

Types of users (higher use among older persons, and women), and use of the different treatment types examined are comparable to that reported by studies in developed countries [4, 5, 7, 14, 18, 26, 34]. Comparison with middle and low income countries is not feasible [8]. However, the rate for psychotherapy use by the total sample (4.6 %), is higher than the 3.2–3.4 % reported recently for the US [26]. This may reflect a general view in São Paulo that CMDs such as depression are psychological and carry less stigma [35], and the availability of providers: psychologists outnumber all other mental health professionals in all areas of Brazil [35, 36].

Direct comparison of sociodemographic characteristics across the three treatment types indicated: (1) that meeting criteria for CMD was not a requirement for receipt of psychotherapy, but (2) it was associated with psychotropic medication use, either alone or with psychotherapy, and (3) not being married was the only characteristic associated with all three modes of treatment.

Psychotherapy, of which there are multiple types which vary in efficacy [9], can be provided by a wide array of therapists, be obtained by payment out-of-pocket, and may not require a diagnosable mental disorder. Although the type of psychotherapy offered may vary by patient symptoms and desire, and insurance that limits payments and requires evidence of need, the field is wide, and given financial means, is readily accessible. Prescribing psychotropic medications, however, as found in other studies including from Brazil [18, 37], while common, is largely restricted to qualified providers (phytotherapy is an exception). Here, 82 % received psychotropic medication from physicians, most commonly psychiatrists. The higher rate of prescribing by psychiatrists may reflect unease by general practitioners with less training and experience in handling mental health disorders [38]. The association of older age with increased odds of psychotropic use, which has been found also in other studies [14, 18], is a matter of concern, since older age is associated with multimorbidity and polypharmacy.

Unmarried status, which may indicate isolation and lack of intimacy, was significantly associated with all treatment types, and has been reported by others [20, 39]. Finally, our data indicate that in São Paulo, where a public/private national health system is present, neither education nor income determined receipt of psychotherapy or psychotropic medication. It is not clear whether attempts to access care varied by socioeconomic status [15, 40] but once in the system, there appears to be equitable use.

### Limitations

This study has several limitations. Information was obtained over a 2-month, rather than a 12-month period, but with little climate change in the area, effect of season of data gathering becomes less relevant. Refusals were replaced by a demographically comparable person, but replacements may nevertheless have differed from those originally selected. Mental health service data were self-reported, and could not be verified. Report of psychotherapy and of psychotropic medications may be underestimated due to problems associated with recall, social desirability, and stigma. The type of psychotherapy received was not determined, but the survey definition of psychotherapy was broad. Unfortunately, information was not obtained on who provided psychotherapy (psychiatrist or psychologist), use of additional informal supports alongside psychotherapy (e.g., self-help, church, family, community healers), or whether psychotherapy and psychotropic medication were obtained from the same provider, or used concomitantly when both were reported.

CMD was assessed by the GHQ-12, which addresses the most prevalent conditions (depression, anxiety, stress-related conditions), but not all (e.g., substance abuse, personality disorders), and no data on alcohol or drug abuse were available. The GHQ-12 is a screen, and does not provide a psychiatric diagnosis, or indicate disease severity. Other major investigations in this field, however, employed the same assessment instrument [15, 16, 41]. Our data refer only to community residents age 18–65. The homeless and those hospitalized or in institutions where CMD rates may be higher, are not represented. While the data were gathered a decade ago, and the Family Health Program and psychiatric services have expanded since then, major changes affecting recognition, referral and treatment of CMD were not planned and are unlikely to have occurred.

## Conclusions

Previous studies have often looked separately at users of psychotherapy and at users of psychotropic medications. Using the same sample, and so keeping the setting constant, this study, uniquely, provides information on CMD, the association of CMD with use of psychotherapy and psychotropic medications, and the characteristics of users of these forms of treatment.

Information from this representative community-resident sample of young and middle-aged adults (age 18–65), indicated that less than a fifth of those who met GHQ-12 criteria for CMD reported using psychotherapy or psychotropic medication in the previous 12 months. In agreement with other studies, a slightly higher proportion who did not meet criteria for CMD used the treatments under study, in particular psychotherapy. An unmarried state appears to carry risks to mental health since it is the only condition common to all three types of intervention. Meeting criteria for CMD increased the odds of receiving psychotropic medication, but was not associated with use of psychotherapy, which may be sought for purposes other than diagnosable psychiatric conditions, and which may be facilitated by the substantial availability of psychologists in Brazil. Use of psychotropic medication was more likely for women, and increased with age. Caution is therefore needed because of an increased likelihood of multimorbidity and polypharmacy.

While this study provides information on the prevalence of CMD, and on the characteristics of users of psychotherapy, psychotropic medications, and both, it does not provide information on outcome. Given the continued expansion of the public health system in Brazil, increased participation by health insurance companies, improved psychotropic medications, and the development of alternative approaches to treatment for CMD, information in this area is essential to advise the public health system with respect to cost effective treatment for patients in need.

## Abbreviations

CMD: common mental disorders
GHQ-12: General Health Questionnaire −12
